# Epigenetic silencing of *MAL*, a putative tumor suppressor gene, can contribute to human epithelium cell carcinoma

**DOI:** 10.1186/1476-4598-9-296

**Published:** 2010-11-22

**Authors:** Wei Cao, Zhi-yuan Zhang, Qin Xu, Qiang Sun, Ming Yan, Jun Zhang, Ping Zhang, Ze-guang Han, Wan-tao Chen

**Affiliations:** 1 Department of Oral and Maxillofacial Surgery, Ninth People's Hospital, Shanghai Jiao Tong University School of Medicine, Shanghai 200011, China; 2 Shanghai Key Laboratory of Stomatology, Shanghai 200011, China; 3 Chinese National Human Genome Center at Shanghai, 201203, China

## Abstract

**Background:**

To identify new and useful candidate biomarkers in head and neck squamous cell carcinoma (HNSCC), we performed a genome-wide survey and found that Myelin and lymphocyte-associated protein (MAL) was a gene that was markedly down-regulated in HNSCC. Hence, we investigated the mechanism of *MAL *silencing and the effects of *MAL *on the proliferation, invasion, and apoptotic potential in HNSCC.

**Results:**

*MAL *was significantly down-regulated in 91.7% of HNSCC specimens at the mRNA level as compared with adjacent normal tissues (*P *= 0.0004). Moreover, the relative transcript levels of the *MAL *gene were remarkably decreased by five-fold in nine HNSCC cell lines as compared with normal head and neck epithelium cells. *MAL *gene expression was restored in 44%, 67%, and 89% in HNSCC cell lines treated with TSA, 5-Aza-dC, and TSA plus 5-Aza-dC, respectively. Furthermore, bisulfate-treated DNA sequencing demonstrated that the two CpG islands (that is, M_1 _and M_2_) located in *MAL *promoter region were completely methylated in the HNSCC cell lines (CpG methylated ratio was more than 90%), and only one CpG island (that is, M_1_) was partially methylated in HNSCC tissues (CpG methylated ratio between 20% and 90%). A significant reduction in cell proliferation and a change in the cell cycle profile were also observed in *MAL *transfectants. Matrigel assay demonstrated that the invasiveness of HNSCC cells significantly decreased. A significant increase in the population of apoptotic cells was observed in *MAL *transfected cells. The exogenous expression of the *MAL *gene suppressed malignant phenotypes, while the cell death induced by *MAL *gene transfer was a result of apoptosis as demonstrated by the induction of cleavage of the poly (that is, ADP-ribose) polymerase. Additionally, tumor growth was suppressed in cells expressing *MAL *as compared with cells not expressing *MAL*.

**Conclusion:**

Our data suggest that the epigenetic inactivation of *MAL*, as a candidate tumor suppressor gene, can contribute to human epithelial cell carcinoma and may be served as a biomarker in HNSCC.

## Background

Head and neck squamous cell carcinoma (HNSCC), which is a type of epithelial carcinoma, is the sixth most common cancer in the world [1]. However, surgery, radiotherapy, and chemotherapy have not sufficiently improved the five-year survival rate of patients with these devastating diseases in more than three decades. Despite ongoing efforts, no biomarkers have been identified yet. To search for useful biomarkers for the diagnosis, therapy, and prognosis of HNSCCs, high-throughput microarray analysis was used to identify genes that are differentially expressed in tumors [2-6]. In a previous study [7], we used an oligonucleotide microarray (Affymetrix HG-U95Av2) to select differentially expressed genes in 22 pairs of HNSCC and normal epithelial tissue from the same donors. Remarkably, the Myelin and lymphocyte-associated protein gene (*MAL*), which is located on chromosome 2q, was down-regulated in HNSCC. The previous reports also described the similar down-modulation of *MAL *gene expression in certain cancer types [8-13], indicating *MAL *gene downregulation might be as a common molecular event contributing to initiation and/or progression of cancers. Based on this hypothesis, *MAL *was chosen for further investigation.

The *MAL *gene was originally identified by Alonso and Weissman during a search for differentially expressed genes in T-cell development [14]. The MAL protein, which is encoded by *MAL*, is also to be a vesicle integral protein. It has a molecular weight of approximately 17 kDa (VIP17) and is present in Schwann cells, in oligodendrocytes, and in the epithelial cells of the kidney, stomach, and large intestine [15]. It spans the membrane four times and is structurally similar to members of the tetraspan family. In every cell in which it is expressed, MAL is associated with glycosphingolipids; it is also thought to be involved in the organization, transport, and maintenance of glycosphingolipid-enriched membranes, which are resistant to detergent extraction. Because these glycosphingolipid-enriched membranes are thought to be part of apical transport machinery [16], MAL as a proteolipid protein is involved in normal apical transport and accurate sorting in Madin-Darby canine kidney cells [17,18]. The recent data on *MAL *gene down-regulation in primary human epithelial malignancies suggest that the loss of the *MAL *gene may be closely linked with a variety of human epithelial malignancies [8,19,20]. Moreover, Beder et al reported that *MAL *gene mRNA is downregulated in metastatic HNSCC tumors as compared to that in primary HNSCC tumors, implying that the decreasing *MAL *expression could contribute to metastatic potential of HNSCC [21]. The use of the MAL protein as a predictive biomarker to benefit for patients undergoing chemotherapy was also statistically analyzed in breast and ovarian cancers [11,22]. However, the molecular mechanism by which of *MAL *gene downregulation and contribution in HNSCC initiation or progression remain unclear. Moreover, whether *MAL *gene might be a valuable diagnostic or therapeutic biomarker for HNSCC need to be further investigated.

In this study, we examined the expression of *MAL *transcripts in HNSCC samples, adjacent normal epithelial tissues and HNSCC cell lines using real-time RT-PCR and semi-quantitative RT-PCR. We found that full-length isoform a is the only transcript variant in head and neck epithelium; *MAL *transcript levels are down-regulated in HNSCC tissues and HNSCC cell lines as compared with adjacent normal tissues. Next, we investigated the mechanism of *MAL *gene down-regulation. The aberrant epigenetic alteration contributes to the silencing of the *MAL *gene in HNSCC. Furthermore, to assess the functional role of *MAL *in HNSCC, we evaluated the effect of ectopic *MAL *expression on Tca cells, Tca-M and Tb cells, which are HNSCC-derived cell lines. Our results show that exogenous expression of *MAL *in HNSCC cell lines inhibited the proliferation, invasion, and induced apoptosis of cancer cells *in vitro *and tumorigenicity *in vivo*.

## Results

### Identification and expression of *MAL *transcript shows variations in head and neck squamous epithelium

**Figure 1:**
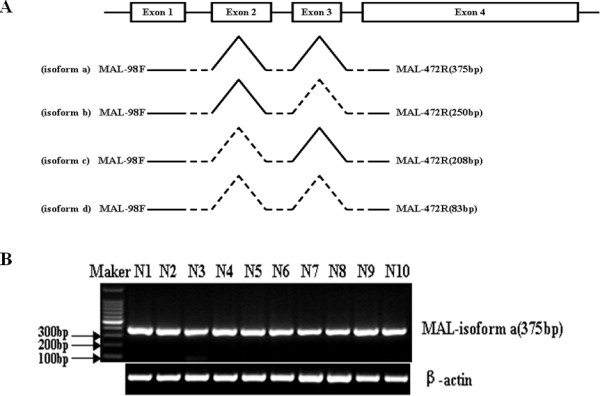
**Identification of *MAL *gene splicing isoforms in oral squamous epithelium**. (A) Structure of the human *MAL *gene and PCR primers used to determine expression of *MAL *variants. The *MAL *gene has four exons and encodes four major transcripts, namely, full-length isoform a and isoform b which lacks exon 3, isoform c which lacks exon 2, and isoform d which lacks exon 2 and exon 3. Primers *MAL*-98F and *MAL*-472R were used to discriminate four variants, namely, a, b, c, and d. (B) Expression of alternatively spliced *MAL *isoforms in 10 cases of normal oral squamous epithelium using semi-quantitative RT-PCR analysis. *β-actin *was amplified as a control.

Alonso et al [23] previously reported that sequence analysis of the human T-cell-specific *MAL *gene revealed four exons, each encoding a hydrophobic, presumably membrane-associated, segment and its adjacent hydrophilic sequence. Alternative splicing produces four transcript variants, which vary by the presence or absence of alternatively spliced exons 2 and 3. To determine whether alternatively spliced isoforms of *MAL *were expressed in head and neck squamous epithelium, a pair of PCR-primers spanning exon 1 and exon 4 was designed to discriminate the transcript levels of the four variants using semi-quantitative RT-PCR analysis (Figure 1A, 1B). Our results showed that the variant a, which is the longest of the isoforms is the only transcript variant in head and neck squamous epithelium.

### *MAL *is frequently down-regulated in HNSCCs and cell lines

**Figure 2:**
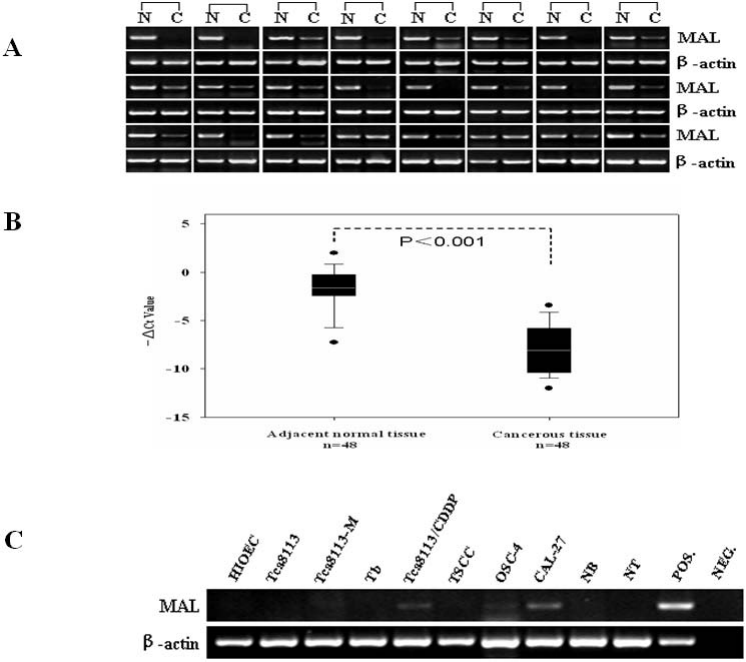
***MAL *mRNA expression in HNSCC specimens and HNSCC cell lines**. (A) Representative results of gel electrophoresis from 24 pairs of HNSCC(C) and their adjacent normal oral epithelium (N). (B) Real-time RT-PCR analysis of *MAL *was carried out in 48 pairs of HNSCC and adjacent normal oral epithelium. For each sample, the relative mRNA level of *MAL *was normalized to β-actin. The line within each box represents the median Ct value; the upper and lower edges of each box represent the 75^th ^and 25^th ^percentiles, respectively; the upper and lower bars indicate the highest and lowest values determined, respectively. (C) Expression of *MAL *mRNA in nine representative HNSCC cell lines and human immortalized oral epithelial cells (HIOEC) using semi-quantitative RT-PCR analysis with normal human oral epithelial cell (NHOEC) as a positive control (POS.) and no cDNA template as a negative control (NEG.).

As our microarray data indicated, *MAL *expression was reduced remarkably in HNSCC as compared to adjacent normal tissues. To investigate whether *MAL *contributes to HNSCC progression, real-time RT-PCR and semi-quantitative RT-PCR analyses were performed to investigate mRNA levels of *MAL *in 48 HNSCC specimens. Out of the 48 cases examined, 44 (91.7%) HNSCC specimens had at least a two-fold decrease in *MAL *mRNA levels as compared to non-tumorous tissues (*P *< 0.01, Figure 2A, 2B). Meanwhile, the expression levels of *MAL *mRNA in a panel of nine HNSCC cell lines and the immortalized oral keratinocyte line HIOEC were detected during semi-quantitative RT-PCR analysis. As shown in Figure 2C, *MAL *transcript levels in the HNSCC cell lines and HIOEC cells were significantly lower than in the case of normal epithelial cells (*P *< 0.01).

### The transcription of *MAL *is associated with promoter methylation in HNSCCs

**Figure 3:**
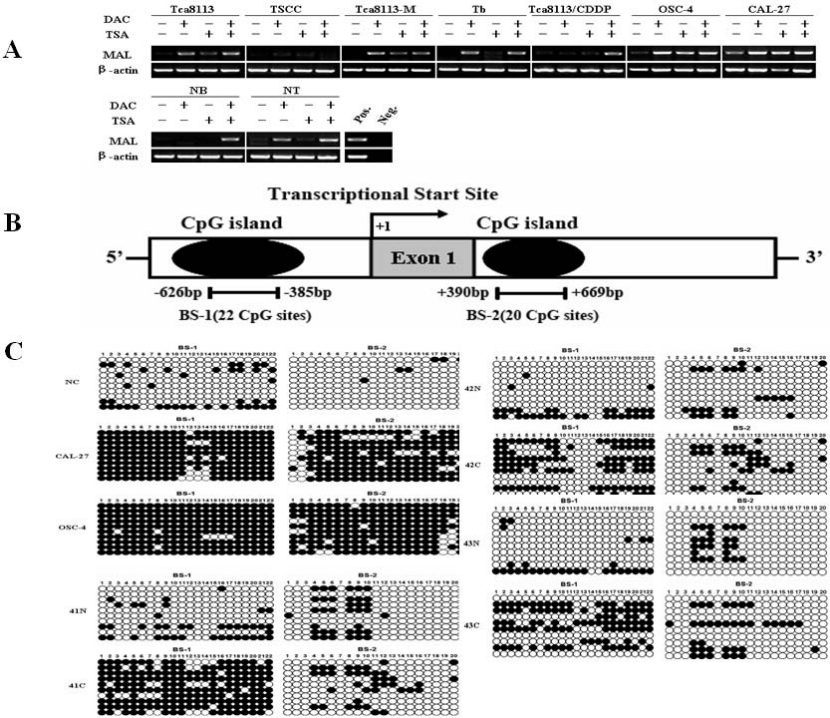
**7Aberrant methylation of promoter region mediated the silencing of *MAL *in HNSCC**. *(*A) Pharmacogenetic treatment of nine HNSCC cell lines with or without 5-Aza-dC and/or TSA. Primary keratinocytes were used as the positive control (POS.), and no template added in the PCR reaction was regarded as the negative control (NEG.). (B) Schematic representations of the location of the CpG islands within the promoter and the intragenic regions of *MAL *and the location of the bisulfate-based sequencing within the CpG island. (C) Representative results from the bisulfate-treated genomic DNA sequencing to detect the methylation status of the BS-1 and BS-2 regions in two representative HNSCC cell lines and seven HNSCCs as compared with the methylation level in primary keratinocytes and matched normal epithelium cells (*P *< 0.05). The numbers indicate the CG dinucleotide within the CpG island in the promoter. Black and white circles represent methylated and unmethylated CpGs, respectively.

Increasing evidence has shown that epigenetic silencing of tumor suppressor genes because of aberrant DNA hypermethylation and histone modification is essential to carcinogenesis and metastasis [20,24]. To verify whether *MAL *gene promoter hypermethylation contributed to *MAL *down-regulation, nine HNSCC cell lines were treated with 5-Aza-dC in conjunction with the TSA. Our result showed that *MAL *gene transcription was reactivated in 67% (6/9) of the HNSCC cell lines after treatment with 5-Aza-dC, while 89% (8/9) of the cell lines showed *MAL *reactivation because of simultaneous treatment with 5-Aza-dC and TSA. In addition, the levels of *MAL *mRNA increased in 44% (4/9) of HNSCC cell lines treated with TSA alone as compared to the untreated control (Figure 3A). To further explore the epigenetic mechanism, the putative CpG islands around *MAL *transcriptional start site (TSS) were screened by using bioinformatics tools. Two major CpG islands M1 (distance to TSS, -626 to -385 bp) and M2 (distance to TSS, +390 to +669 bp) were found. Next, to evaluate the methylation status of the two major CpG islands around the *MAL *TSS, bisulfate-treated DNA sequencing was performed in two randomly selected HNSCC cell lines and seven pairs of HNSCCs from 48 cases. The results demonstrated that both CpG islands (that is, M1 and M2) were completely methylated in the HNSCC cell lines (CpG methylated ratio more than 90%) relative to normal primary head and neck epithelial cells (*P *< 0.01). However, the methylation level of the CpG island (that is, M1) in the *MAL *putative promoter but not the CpG island in the intragenic regions (that is, M2) was significantly higher in all seven HNSCC cases than in the corresponding adjacent non-cancerous tissues (*P *< 0.01, Figure 3B, 3C)

### MAL inhibits cell proliferation and colony formation, thus inducing G1-phase arrest

**Figure 4:**
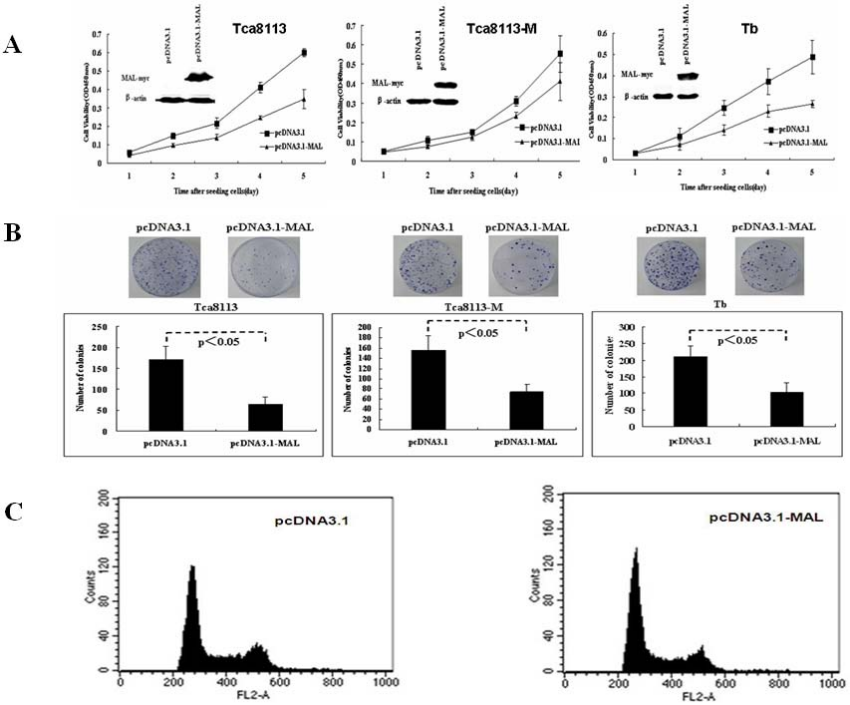
**Effect of MAL on cell growth and colony formation of HNSCC cell lines**. (A) Exogenous *MAL *expressed in Tca, Tca-M and Tb cells transfected with the pcDNA3.1 vector. Parental cells with empty vector were used as a control. The growth of these cells was analyzed using the CCK-8 kit, and the symbols represent the mean values of triplicate tests (mean ± SD). Western blot analysis indicated the expression of MAL in these cell lines. A *t*-test was used to show significant differences between the two groups (*P *< 0.05). (B) To examine the effect of MAL on colony formation, pcDNA3.1-*MAL *was transfected into these three cell lines. After transient transfection for 24 hours, the cells were harvested and seeded onto 100 mm dishes and cultured in G418 for 14 days. The representative dishes show the inhibitory effect of MAL on colony formation, and the lower graphics show that colony formation was significantly suppressed by MAL as compared with the empty vector control (*P *< 0.05). All experiments were repeated at least three times. (C) The cell cycle distribution after transient transfection with pcDNA3.1-*MAL *or an empty vector as assessed by flow cytometry.

To evaluate whether *MAL *functions as a tumor suppressor in HNSCC cells, we assessed the effect of MAL expression on cell proliferation and colony formation. Based on the MAL expression pattern in HNSCC-derived cell lines (Figure 2C), we transiently transfected mammalian expression vectors containing *MAL *into Tca, Tca-M and Tb cells, all of which lacked endogenous MAL expression. MAL over-expression suppressed the growth and colony formation ability of these cells relative to mock-transfected controls (*P *< 0.01, Figure 4A and 4B). To validate which cell population was altered most remarkably in cell cycle distribution, flow cytometry was performed in randomly selected cell line. Our results showed that the *MAL*-transfected cells had a marked increase in the number of cells in the G1-phase as compared with the mock-transfected cells (43.58% ± 5% vs. 59.61% ± 6%, *P *< 0.05, Figure 4C.) This indicates that the cells expressing exogenous MAL become blocked at the G1/S transition.

### Induction of apoptosis in HNSCC cells by ectopic MAL expression

**Figure 5:**
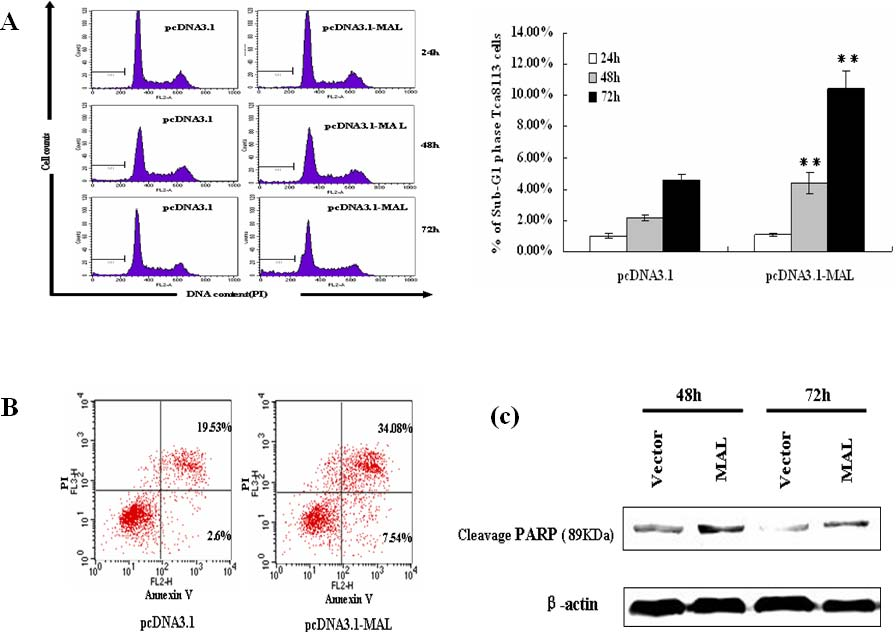
**Effect of MAL on HNSCC cell apoptosis**. (A) Sub-G1 apoptotic cells induced by ectopic expression of MAL in Tca cells transfected with pcDNA3.1-*MAL *vector were analyzed using PI staining and flow cytometry at different time points. Parental cells with an empty vector were used as a control. The lower histogram shows that exogenous MAL markedly induced apoptosis after transient transfection for 48 hours as compared with the control vector (*P *< 0.01). (B) To further observe the effect of MAL on cell apoptosis, after transient transfection for 72 hours, the cells were harvested and stained with 7-AAD and Annexin V-PE and then analyzed using flow cytometry. (C) Expression of cleaved PARP was assessed by Western blotting after transient transfection with pcDNA3.1-*MAL *and the empty vector for 48 hours and 72 hours.

To determine whether the inhibitory effect of MAL expression on cells was caused by apoptosis, two independent assays were performed. As shown in Figure 5A, MAL expression induced an increase in the number of sub-G1 cells. After 24 hours, 48 hours and 72 hours of transient transfection, the apoptotic rates of the *MAL*-transfected Tca cells were 1.14%, 4.04%, and 11.71%, respectively, as compared with 1.07%, 2.20%, and 4.01%, respectively, for the control cells (48 hours and 72 hours, *P *< 0.01). In addition, we assessed the fraction of cells with positive staining for 7-amino-actinomycin (7-AAD) and Annexin V-PE in *MAL*-transfected Tca cells (7.54%) and the control cells (2.60%) (Figure 5B). Furthermore, we also examined the expression level of poly (ADP-ribose) polymerase (PARP) cleavage, which is a hallmark of apoptosis [25], using Western blot analysis after transient transfection with an empty vector or *MAL *at 48 hours, 72 hours (Figure 5C).

### Effect of exogenous MAL expression suppresses invasion of HNSCC cell line

**Figure 6:**
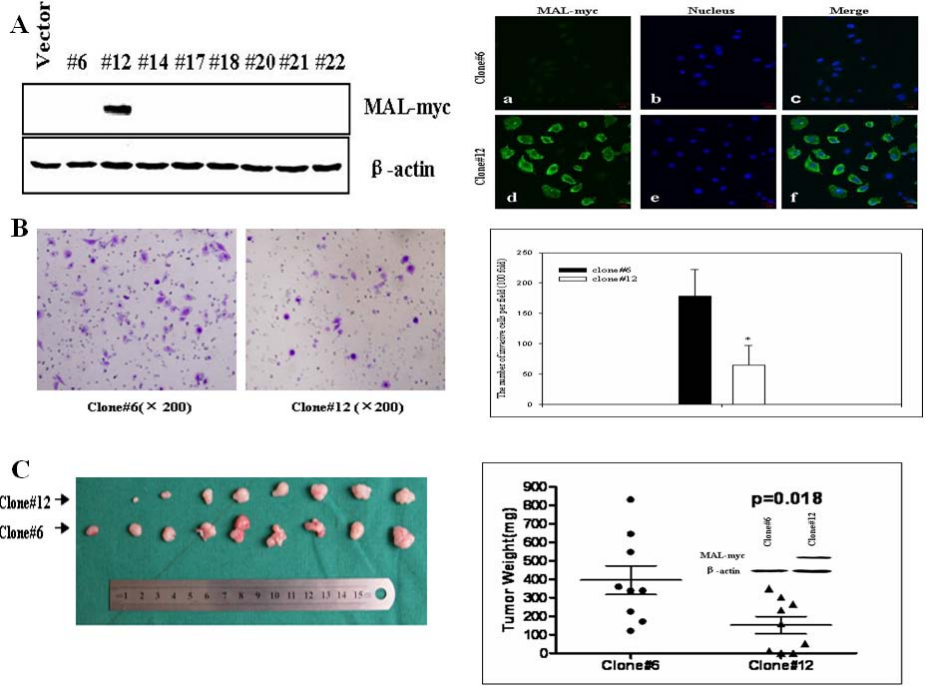
**The effect of MAL on cell invasion and tumorigenicity**. (A) The representative picture shows that stable cells expressing MAL were selected according to Western blotting after the single-cell cluster was cultured in 600 μg/ml of G418 for four weeks, and the stable cells #12 with exogenous expression of MAL were further validated by immunofluorescence as indicated in the lower graphic. (B) Cell invasion of clone #6 and #12 were evaluated with a Matrigel assay. Cell counts represent mean values per field from five fields over three independent experiments (lower) (mean ± SD). (C) Increased ectopic MAL expression inhibited xenograft tumor growth of Tca stable cells #12 relative to Tca stable cells #6 that do not express MAL. These cells were injected subcutaneously into nude mice, and tumors were excised after four weeks. Tumors weights were measured (mean ± SD.), and MAL expression in tumors was assessed using Western blotting.

*In vitro *invasion assays were performed to determine the effect of *MAL *on cell invasion using BD BioCoat™Matrigel™Invasion Chambers. The Matrigel™matrix served as a reconstituted basement membrane *in vitro*. As shown in Figure 6A and 6B, the number of cells that invaded through the transwell membrane in clone #12, which expresses *MAL *(65 ± 32), was significantly lower than those in clone #6, which does not express *MAL *(178 ± 44, *P *< 0.01).

### Ectopic expression of MAL reduces tumor growth in nude mice

Since *MAL *expression represses the malignant properties of HNSCC cells *in vitro*, we also evaluated the effect of *MAL *on the tumorigenicity in a xenograft model. For this purpose, Tca cells expressing *MAL *(clone #12) and cells not expressing *MAL *(clone #6) were injected subcutaneously into the left and right posterior limb of nude mice, respectively. After four weeks, the animals were executed, and the tumor weights were measured. The results showed that tumor growth was significantly reduced in *MAL-*expressing (clone #12) cells as compared to *MAL*-non-expressing (clone #6) cells (*P *< 0.05, Figure 6C).

## Discussion

Several research groups have recently extensively investigated the transcriptome and proteome of HNSCCs using high-throughput microarray technology [26-29]. The aims were to identify genes that were potentially involved in HNSCC progression and to find diagnostic and prognostic gene expression signatures. These molecular signatures could provide key clues to early diagnosis; they could also guide prognosis and the design and monitoring of new therapeutic approaches [30]. In our previous study using cDNA microarray analysis, we identified *MAL *as a remarkably down-regulated gene in HNSCCs as compared with adjacent normal tissues. Furthermore, Lallemant et al [31] reviewed 23 papers published between March 2000 and November 2007 to find that the *MAL *gene was down-regulated in HNSCCs, which is consistent with our microarray data. Thus, we hypothesized that the *MAL *gene could be a candidate HNSCC-specific molecular marker. However, although *MAL *has been implicated in the overall apical delivery of secretory proteins and transmembrane proteins [32,33], the role of *MAL *in HNSCC progression has not been addressed yet. In this study, our data demonstrated a close correlation of MAL expression with carcinogenesis and/or progression of human HNSCC (Figure 2).

The genetic and epigenetic silencing of tumor suppressor genes is considered a vital molecular event in the development and progression of HNSCC [34]. Although *MAL *is located at chromosome 2q13, a region that is involved in large homozygous deletions in juvenile nephronophthisis [35], no deletions or mutations have been reported yet with respect to human cancer. However, the previous studies indicated that DNA methylation level of *MAL *promoter was increased in various types of carcinoma [9,36-38]. Moreover, the induction of *MAL *gene expression by DAC or TSA was described by some studies [8,9,11]. Furthermore, in addition to hypermethylation of *MAL *gene promoter in 9(45%) out of 20 HNSCC cell lines, Beder et al showed that loss of heterozygosity (LOH) still occur in 9 (30%) of 29 primary HNSCCs [21]. Considering these findings, here we hypothesized that *MAL *was inactivated in HNSCC due mainly to the desregulated epigenetic modifications, although not exclude the possibility that the gene dosage of *MAL *such as LOH affects its expression.

To evaluate effect of the DNA methylation and histone acetylation status on *MAL *expression in HNSCC cell lines, in this study, we used pharmacological treatment on these cells with the demethylation agent DAC in conjunction with (and without) the histone deacetylase inhibitor TSA. Consistent with above findings, our results showed that the *MAL *gene was reactivated in most HNSCC cell lines (Figure 3A), suggesting that DNA promoter methylation and histone deacetylation as major factors were involved in silencing the *MAL *gene. Although *MAL *gene hypermethylation in HNSCC cell lines was found by Beder et al [21], more detailed DNA hypermethylation status within *MAL *gene promoter in HNSCC cells, especially in paired HNSCCs and corresponding normal tissues should be further evaluated. Moreover, the correlation between *MAL *transcriptional level and DNA methylation status in these HNSCC specimens needs to be elucidated. To address these issues, we first used bioinformatics tools to determine whether there were CpG islands in the regulatory elements of the *MAL *gene from 2,000 bp upstream to 200 bp downstream of the transcription start site. In addition, we also examined the sequences of the intragenic regions. Interestingly, two major CpG islands were identified. They were located at upstream region, -626 bp to -385 bp (M1, including 22 CpG sites) and downstream region, +390 bp to +669 bp (M2, including 20 CpG sites) of *MAL *TSS (Figure 3B), respectively. It should be pointed out that these CpG islands have never been included in other studies in various cancers [9-11,36,37]. In this study, we employed bisulfate-treated DNA sequencing to measure promoter hypermethylation, which allows for an objective, robust, and global assessment of promoter methylation status as compared with methylation-specific PCR. Our data indicated that both CpG islands (M1 and M2) around the *MAL *TSS were completely methylated in HNSCC cell lines relative to normal primary head and neck epithelium cells (*P *< 0.01). Only the methylation level of the first CpG island (that is, M1) in the *MAL *promoter was significantly higher in all seven HNSCC cases as compared to the corresponding adjacent noncancerous tissues (*P *< 0.01), suggesting M1 is a major hypermethylated region around TSS of *MAL *gene in HNSCC. Accordingly, *MAL *transcript levels were decreased in all seven HNSCC cases as compared to the corresponding adjacent noncancerous tissues. Based these data and findings in other cancers, we proposed that DNA hypermethylation of the *MAL *regulatory elements could be a major contributor to the downregulation of *MAL *in HNSCC. However, it should be emphasized that, in addition to DNA hypermethylation, histone modification such as deacetylation status also affected *MAL *expression in HNSCC. Further study on this issue would provide the novel insight to elucidate the mechanism by which both DNA methylation and histone deacetylation play a synergic role in silencing of *MAL *gene.

MAL was first postulated as tumor suppressor protein by Mimori et al [8]. The reactivated expression of *MAL *gene exerts an inhibitory effect on motility and tumorigenicity of esophageal cancer. Moreover, MAL can induce apoptosis of cancer cells. However, the effect of MAL on HNSCC cells was unclear, due to the lack of *in vitro *and *in vivo *functional experiments for *MAL *gene in HNSCC. Considering the particularity of HNSCC in epidemiology which is remarkably different from other cancer types, as well as the distinct molecule signatures in initiation and progression, in the present work, we evaluated the functional role of *MAL *gene in three HNSCC cell lines in order to determinate whether MAL might be as a therapeutic target for HNSCC. Our experimental results showed that *MAL *gene overexpression could exert strong antiproliferative effects on HNSCC cells by blocking cells at the G1/S transition. Furthermore, the Annexin V-PE and 7-AAD staining, along with the increasing cell population in sub-G1 phase and elevating cleaved PARP level revealed that the inhibitory effect of MAL on cell proliferation was through intensively inducing early apoptosis of cancer cells. Accordingly, our results also demonstrated that exogenous expression of *MAL *could suppress the invasive ability and tumorigenicity of HNSCC cells, which was consistent with the findings described in esophageal cancer.

## Conclusion

Our study shows that *MAL *inactivation is functionally involved in HNSCC progression and that the *MAL *gene promoter is hypermethylated, which is a predictor of the reduced *MAL *mRNA expression levels in both HNSCC tissues and cell lines. As such, our study strongly suggests that the *MAL *gene may be a candidate tumor suppressor in HNSCC and a potential and novel therapeutic target for HNSCC.

## Methods

### Carcinomas and control specimens

The samples were collected at the Department of Oral and Maxillofacial Surgery at the Affiliated Ninth People's Hospital of Shanghai Jiao Tong University School of Medicine from 2000 to 2009. All samples were obtained by surgery and then quickly frozen in liquid nitrogen until DNA and total RNA were extracted. Normal tissues adjacent to the tumors were also obtained with the patient's consent. Tumors were classified histologically and staged according to the tumor-node-metastasis (TNM) classification of malignant tumors [39]. Tumor pathological grade was assessed according to standard criteria [40].

### Cell lines

The human cell lines Tca8113 (Tca), Tca8113-M (Tca-M), Tb, Tca/CDDP (established in the Ninth People's Hospital, Shanghai Jiao Tong University School of Medicine), TSCC (kindly provided by Wuhan University, School of Dentistry, China), OSC-4 (kindly provided by Kochi University, School of Medicine, Japan), NB and NT (kindly provided by Nagasaki University School of Dentistry, Japan) were cultured in RPMI-1640 medium (GIBCO BRL, USA) supplemented with 10% heat-inactivated fetal bovine serum (FBS; GIBCO BRL, NY, USA), penicillin (100 units/ml) and streptomycin (100 μg/ml) at 37°C in a humidified 5% CO2 atmosphere. CAL-27 (American Type Culture Collection, Manassas, VA, USA) was cultured in Dulbecco's modified Eagle medium (DMEM; GIBCO BRL, USA) supplemented with 10% heat-inactivated FBS), penicillin (100 units/ml) and streptomycin (100 μg/ml) at 37°C in a humidified 5% CO2 atmosphere. The immortalized oral keratinocyte line HIOEC from primary normal human oral epithelial cells infected with HPV16E6E7 (established in the Ninth People's Hospital, Shanghai Jiao Tong University School of Medicine) was cultured in a defined keratinocyte serum-free medium (KSFM; GIBCO BRL, USA).

### RNA preparation in semi-quantitive reverse transcription-polymerase chain reaction (RT-PCR) analysis

Total RNA was extracted from each tissue sample and cell line using the TRIZOL reagent (Invitrogen, San Diego, USA). The quality of the total RNA samples was determined by electrophoresis using formaldehyde agarose gels, and the 18 S and 28 S RNA bands were visualized under ultraviolet light. Then 2 μg of total RNA was reverse-transcribed directly to cDNA using a reverse transcription kit (Promega, Madison, USA) following the manufacturer's instructions in a total volume of 25 μl. The primer sequences used were as follows: *MAL *forward, 5'-CAGTGGCTTCTCGGTCTT-3'; *MAL *reverse, 5'-AGCAGAGTGGCTATGTAGGA-3'; *β-actin *forward, 5'-TCACCCACACTGTGCCCATCTACGA-3'; and *β-actin *reverse, 5'-CAGCGGAACCGCTCATTGCCAATGG-3'; Each primer was added at a final concentration of 0.5 μM to a 15 μl reaction mixture in PCR buffer containing 1 μl of cDNA, 0.25 mM of each dNTP, 1.5 mM of MgCl_2_, and 2.5 units of *Taq *DNA polymerase. An initial denaturation was carried out for 5 minutes at 94°C, and 35 cycles were performed with the following PCR program: denaturing at 94°C for 30 seconds, annealing at 45-60°C for 30 seconds for *MAL *and 55°C for 30 seconds for *β-actin*, and elongation at 72°C for 30 seconds. This program was completed with a final extension at 72°C for 5 minutes. Ethidium bromide-stained bands were visualized using UV transillumination, and fluorescence intensity was quantified using the FR-200 system (FuRi, Shanghai, China). The data from semi-quantitive PCR reactions were normalized against the expression of *β-actin *from three independent experiments ± the standard deviation (SD). All RT-PCR data were from at least three independent experiments.

### Real-time PCR

All real-time PCR reactions were performed using a Thermal Cycler Dice™Real Time System (Takara) and the SYBR Premix Ex Taq™reagents kit (Takara, Japan). The real-time PCR was performed in a final volume of 15 μl with 1.5 μl of template cDNA at a concentration of 20 ng/μl with 7.5 μl SYBR green I fluorescent dye and 20 pM of each primer for the target gene and the house keeping gene (*β-actin*). The primer sequences included sense 5'-GGGCTGGGTGATGTTCGT-3' and anti-sense 5'-TAGGCTGCGTCCAAGGTG-3' for *MAL *(117 bp) and sense 5'-CCTGGCACCCAGCACAAT-3' and anti-sense 5'-GGGCCGGACTCGTCATACT-3' for *β-actin*. Results of real-time PCR were represented as Ct values, where Ct was a fraction defined as the cycle number at which the sample fluorescent signal passes a given threshold above the baseline. ΔCt was the difference in the Ct values derived from the specific genes after being assayed and *β-actin*. The N-fold differential expression in a specific gene of a tumor sample, matched normal tissue and cell lines were expressed as 2^ΔCt ^[17]. The significance level was defined as a *P*-value of less than 0.01.

### Induction of gene expression by 5-Aza-dC, TSA and 5-Aza-dC in combination with TSA

To induce demethylation of promoter prior to evaluation for induction of *MAL *expression, all of the above-mentioned HNSCC cell lines were treated with 2 μM 5-Aza-dC, which is the DNA demethylation reagent, for 72 hours, 0.5 μM TSA, which is a specific inhibitor of histone deacetylase, for 24 hours, or 2 μM 5-Aza-dC for 72 hours plus 0.5 μM TSA for 24 hours.

### Direct bisulfate sequencing

Genomic DNA was treated with bisulfate as previously described [18]. Briefly, 1 μg genomic DNA was denatured by incubation with 0.2 M NaOH. Aliquots of 10 mM hydroquinone and 3 M sodium bisulfate (pH = 5.0) were added, and the solution was incubated at 50°C for 16 hours. To analyze the DNA methylation status of the CpG islands of *MAL *promoter in HNSCCs and cell lines, regions enriched in CpG islands were amplified in bisulfate-treated genomic DNA using the primers CpG island I, forward, 5'-GGAGTAATTTTTTATTTTTAGGTAGA-3'; reverse, 5'-AAATTTAAATCTCCTTCATTTTTCC-3'; CpG island II, forward, 5'-TTTAATTGGGGTTAGATGTAGGTAG-3'; and reverse, 5'-AAAAACTTTAAAAAACCAAAAAAAA-3'. The PCR products were then subcloned into a pMD18-T vector (TaKaRa Inc. Japan) for DNA sequencing on an ABI 3730 sequencer.

### Western blotting

The cells were washed with PBS twice and lysed for 30 minutes in 25 mM Tris-HCl with a pH of 7.5 and containing 150 mM NaCl, 5 mM EDTA, and 1% Triton X-100 at 4°C. The lysate was homogenized by passing the sample through a 22-gauge needle. The glycolipid-enriched membrane (GEM) was isolated from the lysate by equilibrium centrifugation for Western blot analysis with an anti-c-myc monoclonal antibody (mAb). In brief, for immunoblot analysis, the GEM samples were run on SDS-PAGE in 12% acrylamide gels and transferred onto nitrocellulose membranes (Amersham, NA, England). After blocking with 5% nonfat dry milk and 0.05% Tween-20 in PBS, blots were incubated with anti-c-myc mAb (Santa Cruz Biotechnology, CA, USA), anti-cleavage PARP polyclonal antibody (Cell signaling, MA, USA) and anti-β-actin mAb (Sigma, St Louis, MO, USA) at the optimized dilutions. Bands were detected using an IRDye™800 Conjugated Affinity Purified Anti-Mouse IgM antibody (Rockland, Gilbertsville, PA, USA). The membrane was then washed several times and scanned using the Odyssey infrared imaging system (LI-COR, Lincoln, NE, USA) at an 800 channel wavelength and analyzed with Odyssey software.

### Plasmid construction and the transient and stable transfection of constructs

To generate the *MAL *expression vector, the open reading frame of human *MAL *cDNA was cloned into the eukaryotic expression vector pcDNA3.1 (Invitrogen, San Diego, CA, USA) and fused to a COOH-terminal Myc tag. The primers used for the amplification of the open-reading frame of the *MAL *cDNA were GCGAATTCACCATGGCCCCCGCAGCGGCGACG (forward primer), with an *EcoRI *site, and GCAAGCTTGCTGAAGACTTCCATCTGATTAAAG (reverse primer), with a *HindIII *site without the stop codon. The amplified *MAL *gene product was purified using a Qiaquick PCR Purification Kit (Qiagen, Chatsworth, CA, USA), cut with *EcoRI *and *HindIII *and ligated with the Ligation Mix (TakaRa, Dalian, China) into the respective *EcoRI *and *HindIII *sites on the eukaryotic expression vector pcDNA3.1. For transient transfection, the cells were transfected with various plasmids at about 80% confluence using the Lipofectamine™2000 (Invitrogen, San Diego, CA, USA) reagent according to the manufacturer's instructions. To generate stable MAL-expressing cell lines, cells were selected with G418 (600 μg/ml) for two weeks, and drug-resistant cells were screened using Western blot analysis with the anti-human c-myc mAb to detect MAL-myc. The clones that exhibited positive immunoreactivity were maintained in G418 (300 μg/ml) medium. Mock-transfected, *MAL*-transfected (but not MAL-expressing) and MAL-expressing stable cells were denoted as 3.1-vector, clone #6, and clone #12, respectively.

### Cell-proliferation assay

Cell-proliferation assay was performed to analyze the proliferation potential of transiently transfected empty vector and *MAL*-transfected cells. To this end, we used the Cell-Counting Kit (CCK)-8 (Dojindo, Kumamoto, Japan). Briefly, the cells were harvested and plated in 96-well plates at 1 × 10^3 ^cells per well and maintained at 37°C in a humidified incubator. At the indicated time points, 10 μl of the CCK-8 solution was added into the triplicate wells and incubated for 1 hour, and the absorbance at 450 nm was measured to calculate the number of vital cells in each well. Measurements were performed in triplicate, and the Mean (±SD) optical density (OD) was reported.

### Colony Formation assay

Twenty-four hours after transfection, the cells (1 × 10^5 ^cells per plate) were plated in 100-mm culture dishes and incubated with 600 μg/ml of G418 for 14 days to allow for colony formation. The colonies were then washed twice with PBS, fixed with 70% ethanol and stained with 0.1% Coomassie Brilliant Blue R-250. Colonies of more than 50 cells were counted under a dissecting microscope. The data from colony formation were shown as means (± SD) from at least three independent experiments, each performed in triplicate.

### Cell cycle distribution assay

Empty vector and *MAL*-transfected cells at the logarithmic growth phase were harvested, and single-cell suspensions containing 1 × 10^6 ^cells were permeabilized with 70% ethanol. The cells were then labeled with 50 μg/ml of propidium iodide (PI) and treated with 250 μg/ml of RNase at 4°C for 30 minutes. Analysis was performed using FACS Calibur (BD Biosciences, San Jose, CA, USA) and analyzed using Cell Quest Software (BD Biosciences, San Jose, CA, USA). The data represent the Means (± SD) from at least three independent experiments.

### Apoptosis assay

The cells were harvested 24 hours, 48 hours, and 72 hours after transient transfection. Then they were washed once in PBS, permeabilized with 70% ethanol for 20 minutes and stored at -20°C overnight. The cells were then subsequently washed with PBS and stained with 50 μg/ml of propidium iodide at 4°C for 30 minutes. Apoptotic cells were assessed by flow cytometry for sub-G1 DNA content. Meanwhile, apoptosis was assessed using Annexin V-PE staining 72 hours after transfection. Then, these cells were quantified by flow cytometry using the Annexin V-PE Apoptosis Detection Kit (BD Biosciences, San Jose, CA, USA) according to the manufacture's protocols. Briefly, floating cells and trypsinized adherent cells were pooled and resuspended in 100 μl of Annexin V-PE Binding Buffer. Then 5 μl of Annexin V-PE and 5 μl of 7-AAD were added, and the cells were incubated for 15 minutes at 25°C. The cells were then resuspended in 400 μl of the Annexin V-PE binding buffer and analyzed immediately by flow cytometry. Then 10,000 events were scored by the FACSCalibur and analyzed using the Cell Quest software.

### Confocal microscope

Stable cells, that is, clone #6 and clone #12, were grown on coverslips and washed once with ice-cold PBS and fixed for 30 minutes in 4% paraformaldehyde at 4°C. For immunostaining, the fixed cells were permeabilized with 0.1% Triton X-100 in PBS for 5 minutes, washed and blocked with PBS containing 0.05% Tween-20 and 5% horse serum (HS) for 30 minutes at room temperature, and incubated with a 1:500 dilution of anti-c-myc mAb in PBS supplemented with 0.05% Tween-20 and 5% HS in a humidified chamber at 4°C overnight. The cells were then rinsed three times with 0.05% Tween-20 in PBS and incubated with a 1:100 dilution of secondary antibody for 30 minutes in 0.05% Tween-20 and 5% HS in PBS. The cells were then rinsed three times with 0.05% Tween-20 in PBS. All fluid was then removed, and two separate drops of Gel Mount-4' and 6' diamino-2-phenylindole·2HCl (DAPI) were applied to the sections. A cover slip was then carefully lowered onto the sections. The cells were then imaged using a TCS-SP laser-scanning confocal microscope with a ×40 oil immersion lens (Leica Microsystems, Mannheim, Germany).

### Cell invasion assay

*In vitro *invasion assay was performed to analyze the invasive potential of stable clone #6 and clone #12 cells. A total of 1 × 10^5 ^cells in 750 μl of serum-free RPMI-1640 medium were plated onto BD BioCoat ™Matrigel ™Invasion Chambers (8 μm pore size; BD Biosciences, USA) with the lower chamber containing 750 μl of RPMI-1640 medium with 10% FBS as a chemoattractant. After 48 hours of incubation in a humidified atmosphere containing 5% CO_2 _at 37°C, the non-invading cells were removed from the upper surface of the membrane by a cotton swab. The membranes were then fixed with methanol and stained with 0.5% crystal violet stain. Invading cells were photographed and counted in five random non-overlapping 200× fields under a light microscope.

### Tumorigenicity *in vivo*

Nude mice (4-6 weeks old) were used for xenograft studies. Tca cells (1.5 × 10^6 ^in PBS) expressing MAL (clone #12) or not (clone #6) were injected subcutaneously in the extremities of the nude mice. After four weeks, nude mice bearing tumors were executed, and tumor weight was measured using an electronic balance. The values for nine mice in each group were averaged to obtain the mean ± (SD) Differences in tumor weights were analyzed using the Student's *t*-test.

### Statistical analysis

Semi-quantitative RT-PCR, real-time PCR and *in vitro *statistical analyses were performed with software from SPSS 13.0 for Windows (Chicago, IL, USA). Results of the semi-quantitative RT-PCR and real-time PCR analyses were evaluated using the Mann-Whitney test for two independent groups. The results of the cell proliferation assay, colony formation assay, and *in vitro *invasion assay were evaluated using Student's *t*-tests. A *P*-value of less than 0.05 was considered significant.

## Competing interests

The authors declare that they have no competing interests.

## Authors' contributions

WTC, ZGH, and ZYZ were responsible for the study design, interpretation of the data and revision of the manuscript. WC responsed for the study of growth inhibition *in vitro *and *in vivo*, gene overexpression, cell apoptosis and methylation assay. WC, PZ, and MY were responsible for data acquisition, analysis of the work presented and the preparation of the draft of the manuscript. JZ, QX, and QS helped to obtain the tissues and to do the experiment *in vivo*. WTC, ZGH, and ZYZ supervised the studies and helped to revise the manuscript. All authors read and approved the final manuscript.
